# External beam radiation therapy versus radical prostatectomy for high-risk prostate cancer: protocol of the RECOVER study

**DOI:** 10.1186/s12885-025-13511-7

**Published:** 2025-01-27

**Authors:** Caroline M. van der Starre, Chris H. Bangma, Maarten J. Bijlsma, Alfons C.M. van den Bergh, Lambertus A.L.M. Kiemeney, Wietske Kievit, Kees Vos, Diederik M. Somford, Sally M. Wildeman, Katja K.H. Aben, Igle J. de Jong, Floris J. Pos, Berdine L. Heesterman

**Affiliations:** 1 Netherlands Comprehensive Cancer Organisation, Utrecht, the Netherlands; 2 Department of Urology, University Medical Center Groningen, Groningen, the Netherlands; 3 Department of Urology, Erasmus University Medical Centre, Rotterdam, the Netherlands; 4 PharmacoTherapy, -Epidemiology and -Economics, Groningen Research Institute of Pharmacy, University of Groningen, Groningen, the Netherlands; 5 Radiation Oncology, The Netherlands Cancer Institute, Amsterdam, the Netherlands; 6 Department IQ Health, Radboud university medical center, Nijmegen, the Netherlands; 7 Patient Representative, Dutch Prostate Cancer Foundation (Prostaatkankerstichting), Utrecht, The Netherlands; 8 Department of Urology, Canisius-Wilhelmina Hospital, Nijmegen, the Netherlands; 9 Prosper Prostate Cancer Clinics, Nijmegen/Eindhoven, The Netherlands; 10 Department of Urology, Franciscus Gasthuis en Vlietland, Rotterdam, the Netherlands

**Keywords:** Prostate cancer, Patient reported outcomes, Health-Related Quality of Life, Prostatectomy / methods, Prostatic neoplasm / radiotherapy, Prostatic neoplasms / surgery, Androgens, Cost effectiveness, Progression free survival, Distant metastasis free survival.

## Abstract

**Background:**

This paper describes the rationale and design of the RECOVER study. Currently, there is no consensus regarding the optimal treatment for high-risk, non-metastatic prostate cancer (PCa). The study primarily aims to evaluate and compare the impact of treatment with robot-assisted radical prostatectomy (RP) versus external beam radiation therapy (EBRT) with androgen deprivation therapy (ADT) for men with high-risk, non-metastatic PCa regarding health-related quality of life (HRQoL) and functional outcomes. Secondary objectives are progression-free survival (PFS), distant metastasis-free survival (DMFS), costs and cost-effectiveness.

**Methods:**

The RECOVER study is a comparative effectiveness study that prospectively includes newly diagnosed high-risk (cT3a-bN0M0, ISUP-grade ≥ 4 and/or PSA > 20 ng/mL), non-metastatic PCa patients. Four Dutch prostate cancer networks, comprising 29 hospitals, are currently participating in the study. Patient reported outcomes are collected before treatment initiation, 12 months and 36 months after treatment initiation and include the EORTC-QLQ-C30, the EPIC-26, an adapted version of the SCQ, an adapted version of the iMTA Productivity Cost Questionnaire and several specific questions regarding patient characteristics, treatment of PCa specific complaints and health resources used. Clinical data regarding patient-, tumor- and treatment characteristics and oncological outcomes are collected up to 5 years after diagnosis. For sufficient power, patient reported outcomes of 471 patients must be collected 36 months after treatment initiation. Descriptive statistics and mixed-effects models are used to assess differences in HRQoL and functional outcomes over time between the patients treated with radical prostatectomy versus EBRT (+ ADT). Inverse probability of treatment weighting or the g-formula are used to adjust for confounding covariates associated with treatment. Secondary endpoints PFS and DMFS are evaluated using a competing risk analysis and cost-utility and budget-impact analyses will be performed to determine cost and cost-effectiveness.

**Discussion:**

An observational prospective design was chosen since a randomized controlled trial comparing surgery and radiotherapy was not deemed feasible. This study evaluates effectiveness of treatment in a routine clinical setting (with adjustment for confounding) and its findings will enhance patients’ and healthcare professionals’ awareness for the impact of both treatment modalities on (long-term) daily functioning and HRQoL and aid treatment decision making.

**Trial registration:**

This study is registered at ClinicalTrials.gov (NCT05931419).

## Background

Approximately 15–18% of all prostate cancer diagnoses concern high-risk, non-metastatic prostate cancer (PCa) [1–3]. High-risk PCa is a heterogenous disease with increased risk of biochemical recurrence, metastatic progression and death from PCa as compared to low- or intermediate-risk localized disease [4–6]. Treatment modalities widely used for high-risk PCa are (robot-assisted) radical prostatectomy (RP) and external beam radiation therapy (EBRT), the latter generally combined with androgen deprivation therapy (ADT). Currently, there is no consensus regarding the optimal treatment for high-risk PCa [7]. This is also reflected in the substantial interhospital treatment variation in the Netherlands [8].

High-level comparative evidence regarding the optimal treatment for high-risk PCa is lacking. Several non-randomized studies have compared oncological outcomes between treatment with RP versus EBRT. Most were retrospective in nature and were performed in a single-institution. These studies did not show clear differences in five-year prostate cancer specific survival (PCSS) and overall survival (OS) [9–14]. Due to variation in the definition of high-risk prostate cancer and differences in applied surgical and radiotherapy treatment strategies, definitive conclusions cannot be drawn [9]. However, survival was generally good with reported five-year OS and PCSS rates exceeding 90% [9–14]. In a recent systematic review we summarized evidence for patient reported functional outcomes and HRQoL after RP compared to EBRT for high-risk, non-metastatic PCa [9]. Three observational studies focused on differences in functional outcomes and HRQoL between RP and EBRT with ADT for high-risk PCa. Overall, sexual dysfunction and genitourinary toxicity were reported more often after RP, while reduced hormonal function and gastro-intestinal toxicity were more common after EBRT combined with ADT [12, 15, 16]. However, due to the limited number of studies, the variation in the definition of high-risk PCa and the differences in methods and measurement instruments, the extent of the effect of both treatment modalities on functional outcomes and HRQoL remains largely unknown. Hence, it remains uncertain whether either treatment is superior with respect to functional outcomes and HRQoL as well as oncological outcomes.

Here we present the RECOVER study (High-Risk prostatE Cancer radiatiOn Versus surgERy) as a means to address these knowledge gaps. The study is primarily designed to evaluate and compare the impact of treatment with RP versus EBRT with ADT on functional outcomes and HRQoL for men with high-risk, non-metastatic PCa. Our study aims to enhance awareness of both patients and healthcare professionals regarding these effects of treatment. By incorporating our findings into a patient decision aid, it could support patients in making a well-informed treatment decision. Moreover, providing insight into the impact of both treatments could lead to a reduction in interhospital and inter-physician variability and improvement in quality of care facilitated by the adjustment and/or refinement of treatment recommendations concerning this patient population in (inter)national guidelines.

## Methods

### Design and collaboration

The RECOVER study is a comparative effectiveness study that prospectively includes patients with high-risk, non-metastatic PCa to compare treatment with robot assisted radical prostatectomy (RARP) versus EBRT combined with ADT. The study is executed by a cooperation of the Netherlands Comprehensive Cancer Organization (IKNL), the Dutch Association for Urology (NVU) and the Dutch Society of Radiotherapy and Oncology (NVRO). The study is performed in close collaboration with the Dutch Prostate Cancer Foundation (Prostaatkankerstichting [PKS]). Currently, 29 hospitals collaborating in four Dutch prostate cancer networks participate in the study, collectively covering 40% of all newly diagnosed prostate cancer patients in the Netherlands. ClinicalTrials.gov provides an up-to-date overview of all participating hospitals [17]. Healthcare providers from hospitals in a prostate cancer network discuss patient, tumor and other relevant characteristics in multidisciplinary team meetings (MDTMs) to achieve the most optimal treatment based on evidence-based guidelines and tailored to the patient. For this study, eligibility of a patient for treatment with both RARP and EBRT with ADT is discussed in these meetings. The participating networks are geographically distributed across the Netherlands and each network consists of at least one university hospital and several non-university teaching hospitals and general hospitals. In the Netherlands, surgical treatment of PCa is centralized in 14 hospitals that meet the minimum hospital volume norm of 100 RARP procedures per year [18]. Within each prostate cancer network, all RARP treatments are centralized in one hospital. Radiation therapy is centralized to 20 institutions across the Netherland and is performed in nine of the participating hospitals.

### Patient population

Patients with histologically confirmed high-risk, non-metastatic PCa diagnosed or treated in one of the participating hospitals are eligible for inclusion. Various (inter)national risk classifications and guidelines define high-risk PCa with subtle differences. In this study, high-risk, non-metastatic PCa is defined as cT3a-bN0M0 disease, and/or ISUP grade ≥ 4 and/or a PSA value at diagnosis above 20 ng/mL. Prostate cancer is staged according to the 8th edition of the Union for International Cancer Control (UICC) TNM classification with the exception that T-stage will be based on either digital rectal examination (DRE) or magnetic resonance imaging (MRI), whichever is highest [19, 20]. Patients must be aged 50 to 75 years, fit (WHO performance status 0–1) and eligible (i.e. no contra-indications) for both RARP and ERBT with ADT. Moreover, patients have to reside in the Netherlands and be able to read and understand Dutch language. Patients with histological PCa types other than adenocarcinoma and those that are diagnosed or treated in a hospital abroad are excluded. Patients treated with androgen receptor signaling inhibitor (ARSI)/androgen receptor targeted agents (ARTA) as part of the initial treatment plan are also excluded.

### Treatment

RARP may be part of a multimodal therapy with (neo)adjuvant ADT and/or adjuvant radiotherapy. A pelvic lymph node dissection (PLND) may be performed for staging purposes. The presence of positive lymph nodes (pN1) upon PLND is not a reason for exclusion and may be followed by adjuvant treatment e.g. lymph node irradiation and/or ADT.

Regarding EBRT, the study allows for dose-escalation using intensity modulated radiation therapy (IMRT), volumetric modulated arc therapy (VMAT) or stereotactic radiation. Dose-escalation is defined as a biologically effective dose (BED) converted to 2 Gy fractions (using a α/β ratio of 1.5) of at least 76 Gy. Both conventionally fractionated and hypofractionated regimens may be used and patients may receive an integrated boost to visible intraprostatic lesions, a low dose rate (LDR) brachytherapy boost or a high dose rate (HDR) brachytherapy boost. In addition, treatment with ADT should be given for a period of at least 6 months. Similar to radical prostatectomy, PLND may be performed for staging purposes and pN1 is no reason for exclusion.

### Study endpoints

The primary endpoints of the RECOVER study are functional outcomes and HRQoL 36 months after treatment initiation. These endpoints are measured with the validated Dutch versions of the Expanded Prostate Cancer Index Composite-26 (EPIC-26) [21] and the European Organization for Research and Treatment of Cancer Quality of Life Questionnaire-C30 (EORTC-QLQ-C30) [22]. We assume that the long-term effects of treatment, including the effects of ADT (patients eligible for inclusion generally receive ADT for 6 to 24 months), have stabilized 36 months after treatment initiation.

Secondary study endpoints are progression-free survival (PFS), distant metastasis-free survival (DMFS), cost and cost-effectiveness. Cost and cost-effectiveness will be evaluated 36 months after treatment initiation. PFS and DMFS are evaluated at 5 years after diagnosis.

### Patient inclusion

Eligible patients are informed about the study by their physician (or research nurse/nurse specialist) shortly after diagnosis. Study information is provided to the patient after which the patient has time to decide whether to participate in the study. Patient information is set up in such a way that it appeals to patients of diverse backgrounds. Patients willing to participate provide written informed consent and administrative data are entered in an online registration tool. Inclusion is finalized when the patient completes the first questionnaires before treatment initiation (baseline measurement). In some hospitals, the EPIC-26 and EORTC-QLQ-C30 are administered as part of the regular care. To minimize patient burden, these questionnaires are reused if possible.

### Data collection

#### Patient reported outcome measures (PROMs)

The EPIC-26 is an instrument developed and validated for the comprehensive assessment of HRQoL in men with PCa [21]. It is one of the best rated instruments for this purpose [23] and consists of 5 symptom domains: urinary incontinence, urinary irritative/obstructive, bowel, sexual, and hormonal. Questionnaire items are converted to domain scores between 0 and 100, with a higher score representing a better function. Threshold values for a clinically meaningful change (minimal clinically important difference [MCID]) have been determined for each EPIC-26 domain: 6 to 9 for urinary incontinence, 5 to 7 for urinary irritation, 4 to 6 for bowel function, 10 to 12 for sexual function and 4 to 6 for hormonal function [24].

The EORTC-QLQ-C30 is a widely used measurement instrument to assess HRQoL of cancer patients [22]. The questionnaire includes five functional domains (physical, role, cognitive, emotional and social), three symptom domains (fatigue, pain, nausea and vomiting) a global health/quality of life scale and several single-item measures. For each domain and single item measure, a score from 0 to 100 can be calculated. For the functional domains and global score, higher scores indicate a better quality of life. In contrast, for the symptom domains and single item measures, a higher score represents a higher level of symptomatology [22]. Musoro et al. have provided minimal important differences for within-group change and between-group change per EORTC QLQ-C30 scale for nine cancer types, amongst which prostate cancer [25].

In addition, patients are asked to complete an edited version of the self-administered comorbidity questionnaire (SCQ) and an adapted version of the Institute for Medical Technology Assessment Productivity Cost Questionnaire (iMTA PCQ). The SCQ is a generic questionnaire developed to assess common comorbidities which might impact an individual’s functioning. For each condition, the presence, severity and functional limitation is self-assessed [26]. We have extended the SCQ with conditions that are considered a (relative) contra-indication for RARP or EBRT, e.g. inflammatory bowel disease.

The iMTA PCQ is a standardized questionnaire developed to measure and value productivity loss. Productivity loss is measured in three modules being absenteeism, presenteeism and productivity loss of unpaid work [27]. The iMTA PCQ was slightly adapted in collaboration with an expert in health technology assessment by focusing questions regarding unpaid work on volunteer work only. Moreover, we reformulated some questions to make them more easily understandable for the patient. The iMTA PCQ is used to determine indirect non-medical costs.

Finally, information is collected regarding patient characteristics, such as living situation, alcohol and smoking behavior and education level, and used health resources for prostate-cancer related problems such as erectile or urinary complaints (e.g. urinary pads, catheter, medication). Information regarding used health resources is collected to determine healthcare costs. Table 1 presents the collected PROs at the various measurement moments.

The patient can complete either paper or web-based questionnaires based on their preference. Web-based questionnaires are collected by use of the Profiles Registry (Patient Reported Outcomes Following Initial treatment and Long term Evaluation). Profiles is specialized in collecting, processing and storing PROs of cancer patients [28] and all data are handled according to the General and Dutch Data Protection Act. Data from the PROFILES registry can be linked to the Netherlands Cancer Registry (NCR), which allows the merging of PROs and clinical data [29]. In order to achieve good response rates, a research assistant monitors the questionnaire response. Patients with incomplete questionnaires will be contacted and information will be complemented as far as possible.

**Table 1:** Overview of the collection of patient reported outcomes in the RECOVER study at the subsequent measurement moments (baseline, T12 and T36)

Category	Included topics / patient reported outcome measures	Baseline	T12	T36
Patient characteristics	Age, length, height, living situation, alcohol and smoking behavior, education level, participatory decision making style	X		
Comorbidities	Adapted version of the SCQ	X	X	X
HRQOL and functional outcomes	EPIC-26 and EORTC-QLQ-C30	X	X	X
Treatment of PCa specific complaints / additional treatment information	Catheter, medication for lower urinary tract symptoms, pelvic floor physical therapy, surgery for urinary loss, erectile medication, use of vacuum pump erectile device, androgen deprivation therapy	X	X	X
Productivity loss	Adapted version of the iMTA PCQ	X	X	

#### Clinical data

In addition to PROs, clinical data regarding patient and tumor characteristics, as well as treatment information, is needed to meet our research objectives. These data are extracted from the Netherlands Cancer Registry (NCR). Maintained by the Netherlands Comprehensive Cancer Organisation (IKNL), the NCR is a nationwide population-based registry that contains information on all newly diagnosed cancer patients in the Netherlands. Clinical data are retrieved from patients electronic health records in the hospitals by well-trained, independent data managers of the NCR. The standard data collection in the NCR is expanded for patients included in this study and includes patient characteristics (i.e. age, height, weight, comorbidities), tumor characteristics (i.e. TNM staging, PSA at diagnosis and Gleason grade on biopsy and prostatectomy if applicable), diagnostic procedures (i.e. imaging, biopsy technique, pelvic lymph node dissection) and treatment information for prostatectomy (i.e. nerve sparing procedure, positive margins, complications) and EBRT (i.e. type of radiotherapy, dose per fraction, integrated/brachytherapy boost, toxicity). In addition, data is collected on all post-treatment PSA measurements, disease progression, subsequent treatments and cause of death until 5 years after diagnosis. Vital status of all patients is updated annually by linkage to the personal records database (BRP) which contains data on the vital status, date of death and emigration status of all Dutch citizens.

Moreover, additional clinical data related to volumes of care are collected from the electronic health records to evaluate healthcare costs. In addition to the treatments for prostate cancer, these data include treatments for adverse events, consultations with medical specialists, hospitalizations and medication use.

### Statistical analysis and sample size

Descriptive analyses will be used to provide insight in patient and clinical characteristics of the total study population and of both treatment groups. Mixed-effects models will be used to compare functional outcomes and HRQoL between the two treatment groups over time. Differences in functional outcomes and HRQoL between treatment modalities as measured by the EPIC-26 will be considered clinically meaningful if they are equivalent to or exceed the MCID and are statistically significant at the 0.05 level. The p-value maintained for statistical significance will be adjusted for multiple testing if appropriate. For the EORTC-QLQ-C30, the between-group anchor-based minimally important differences per scale as reported by Musoro et al. will be used to compare treatment groups over time. For scales for which no anchor-based estimates are available, distribution-based estimates will be used [25]. Due to the observational design of the study, we will adjust for confounding covariates associated with treatment with RARP versus EBRT (and ADT) using inverse probability of treatment weighting or the g-formula [30–33]. In case of missing values in covariates, multiple imputation procedures will be used [34, 35]. Competing risk survival analyses will be used to evaluate PFS and DMFS with the competing event being a non-PCa related death.

To evaluate incremental cost-effectiveness of EBRT combined with ADT versus RARP over a time horizon of 36 months, a cost-utility analysis (CUA) will be conducted both from a societal and a medical perspective. Costs are assessed by multiplying the voluminal with the unit prices. Utilities will be derived by means of a mapping algorithm from the EORTC QLQ-C30 at each time point. The derived utility will be used for the estimation of a quality adjusted life year (QALY) according to the trapezium rule. Incremental costs between treatments will be related to incremental QALYs in a cost-utility ratio (ICUR) to determine the additional costs that need to be spent to gain one QALY. Subsequently, a budget impact analysis (BIA) will be performed to predict the financial consequences related to the adaption and implementation of the favored strategy based on the cost-effectiveness analysis.

Assuming that the effect of treatment on HRQoL is predominantly determined by functional outcomes, the sample size was calculated to demonstrate the mean or upper limit of the MCID-range for each domain of the EPIC-26. A two sided T-test (alpha = 0.05, power 80%) using expected standard deviations, derived from previous research evaluating the impact of RP versus EBRT and ADT on functional outcomes, yielded a required total response of 471 patients at T36 [12]. To account for confounding factors, the sample size was increased by 15%. Based on previous experience with collecting PROs, we assume that 75% of men who are willing to participate in the study and complete the baseline questionnaire will do so again at each subsequent measurement moment. Therefore, 837 patients need to be included in the study (response at T0) for sufficient power.

### Patient and public involvement

The Dutch Prostate Cancer Foundation (Prostaatkankerstichting, [PKS]) was involved in the design of this study. A patient representative of the foundation is part of the RECOVER study steering committee and therefore involved throughout the whole study. Next to this, PKS facilitates the communication of study-related information and research findings via their channels to patients.

### Ethical approval and consent to participate

The Medical Research Ethics Committee Oost-Nederland declared that the study is not subject to the Medical Research involving Human Subjects Act and Medical Treatment Contracts Act in June 2022 (2022–13682). An informed consent is filled out by all patients willing to participate in the study. Moreover, this study was approved by the Privacy Review Board of the NCR (k23.0170).

## Results

### Start-up and patient accrual

Patient recruitment is set up in a step-wise fashion; patient accrual started in February 2023 in the first prostate cancer network (including 3 hospitals), followed by the second network (6 hospitals) in June 2023. The third network (8 hospitals) started patient accrual in October 2023 and the final network (11 hospitals) started in January 2024. The study might be expanded toadditional hospitals during the inclusion period. An up-to-date overview of the participating hospitals is accessible from ClinicalTrials.gov [17]. We anticipate that patient accrual will be completed by 2026, collection of PROs will be finalized by 2029 and collection of clinical follow-up data by 2031. Currently (January 2025), 4 prostate cancer networks and 340 patients participate in the RECOVER study.

## Discussion

RARP and EBRT (often combined with ADT) are frequently used treatment modalities for high-risk, non-metastatic PCa. Research is needed to provide further insight into differences in functional outcomes, HRQoL, oncological outcomes and costs. By evaluating and comparing both treatments we aim to enhance patients’ and healthcare professionals’ awareness regarding the impact of both treatment modalities and aid treatment decision making. Moreover, our results could contribute to a reduction in inter-hospital and inter-physician variability and an improvement in the quality of care, facilitated by the adjustment and/or refinement of treatment recommendations concerning this patient population in (inter)national guidelines.

When designing the RECOVER study, a prospective observational design was purposely chosen over a randomized controlled trial. Randomized controlled trials are the mainstay and gold standard for efficacy studies as they can provide compelling evidence for cause-effect relationships, have the potential for great internal validity and minimize confounding. Patient accrual, however, has shown to be difficult for trials randomizing over surgical and radiation based treatment arms for PCa [36–38]. Both patients and their treating physicians often have a preference for treatment with RARP or EBRT and patients do not want to be randomly assigned to a treatment [36, 39]. As low patient accrual can lead to early termination of the study and consequently no or inconclusive results, a RCT was not deemed feasible. A prospective comparative effectiveness cohort study is the next best alternative to compare RARP and EBRT [40, 41]. Observational study designs evaluate effectiveness of treatment rather than efficacy, are more ethically feasible, reflect daily practice more closely and therefore have good generalizability and can enroll more patients due to lower costs [42, 43]. This enhances statistical power and enables the possibility to generalize results to the high-risk, non-metastatic PCa patient population at large [43]. A limitation of observational studies is the risk of confounding. We aim to improve comparability between both treatment groups by means of the inclusion criteria (e.g. age, performance status). Furthermore, detailed information regarding possible confounding factors will be collected in both our clinical data and questionnaires. In our analyses, measured baseline covariates are balanced across treatment groups with inverse probability of treatment weighting or by covariate adjustment using the g-formula to create comparable populations.

Through its pragmatic design, the RECOVER study accommodates variability in current clinical practice. Surgical treatment may be part of a multimodal strategy with (neo)adjuvant ADT and/or adjuvant radiotherapy. Variation in the RARP group is expected to be limited as (neo)adjuvant ADT is not commonly applied in the Netherlands and early salvage radiotherapy is often preferred over adjuvant radiotherapy even for pN1 disease. In current clinical practice, most patients treated with EBRT (> 90%) receive adjuvant ADT with a LHRH agonist for the duration of 6 to 36 months. In a recent Dutch consensus meeting, the duration of ADT for high-risk PCa was proposed to be 6 to 24 months depending on tumor characteristics.

Besides QoL-related and oncological endpoints, our study will provide insight into the costs and cost-effectiveness of both treatment modalities. Costs of healthcare have been rising substantially over the past decades. A significant proportion of these costs is directed at cancer care, of which prostate cancer is indicated as one of the priciest cancers [44]. In order to control the rising healthcare expenses, it is important to consider costs and cost-effectiveness of treatment. Only few studies have been performed regarding the costs of RP and EBRT for high-risk, non-metastatic PCa and these studies mostly focused on the American or Canadian health care system. This study will provide insight into the cost and cost-effectiveness of care for high-risk, non-metastatic PCa from a Dutch societal and medical perspective.

Altogether, we aim to provide insight into the effects of treatment with RARP versus EBRT combined with ADT from a multidimensional view, including functional outcomes, HRQoL, PFS, DMFS and cost-effectiveness. Thereby we aim to create opportunities for the improvement of quality of life and quality of care for patients with high-risk, non-metastatic PCa.

## Data Availability

No datasets were generated or analysed during the current study.

## Abbreviations

ADT: Androgen deprivation therapy
ARSI: Androgen receptor signaling inhibitor
ARTA: Androgen receptor targeted agents
BED: Biologically effective dose
BIA: Budget impact analysis
BRP: Personal records database
CTCAE: Common toxicity criteria for adverse events
CUA: Cost-utility analysis
DMFS: Distant metastasis free survival
DRE: Digital rectal examination
EBRT: External beam radiation therapy
EORTC-QLQ-C30: European Organization for Research and Treatment of Cancer Quality of Life Questionnaire-C30
EPIC-26: Expanded prostate cancer index composite-26
HDR: High dose rate
HRQoL: Heath-related quality of life
ICUR: Incremental cost-utility ratio
IKNL: Netherlands Comprehensive Cancer Organisation
IMRT: Intensity modulated radiation therapy
iMTA: Institute for medical technology assessment
LDR: Low dose rate
MDTM: Multidisciplinary team meetings
MREC: Medical review ethics committee
MRI: Magnetic resonance imaging
NCR: Netherlands Cancer Registry
NVRO: Dutch Society of Radiotherapy and Oncology
NVU: Dutch Association for Urology
OS: Overall survival
PCa: Prostate cancer
PCSM: Prostate cancer-specific mortality
PCSS: Prostate cancer-specific survival
PCQ: Productivity cost questionnaire
PFS: Progression-free survival
PKS: Dutch PCa patient foundation
PLND: Pelvic lymph node dissection
PROFILES: Patient reported outcomes following initial treatment and long term evaluation
PROs: Patient reported outcomes
PROMs: Patient reported outcomes measures
PSA: Prostate specific antigen
RARP: Robot-assisted radical prostatectomy
RECOVER: High-Risk prostatE Cancer radiatiOn Versus surgERy
RP: Radical prostatectomy
SCQ: Self-administered comorbidity questionnaire
VMAT: Volumetric modulated arc therapy
QALY: Quality adjusted life year

## References

[1] Dee EC, Nezolosky MD, Chipidza FE, Arega MA, Butler SS, Sha ST, et al. Prostate cancer-specific mortality burden by risk group among men with localized disease: implications for research and clinical trial priorities. Prostate. 2020;80(13):1128–33. 32659024 10.1002/pros.24041

[2] Cooperberg MR, Broering JM, Carroll PR. Time trends and local variation in primary treatment of localized prostate cancer. J Clin Oncol. 2010;28(7):1117–23. 20124165 10.1200/JCO.2009.26.0133 PMC2834465

[3] Netherlands comprehensive cancer organisation (IKNL). Prostaatkanker in Nederland Een overzicht op basis van de Nederlandse Kankerregistratie (1989 tot en met 2020) [Available from: https://iknl.nl/prostaatkanker-in-nederland].

[4] D’Amico AV, Whittington R, Malkowicz SB, Schultz D, Blank K, Broderick GA, et al. Biochemical outcome after radical prostatectomy, external beam radiation therapy, or interstitial radiation therapy for clinically localized prostate cancer. JAMA. 1998;280(11):969–74. 9749478 10.1001/jama.280.11.969

[5] Boorjian SA, Karnes RJ, Rangel LJ, Bergstralh EJ, Blute ML. Mayo Clinic validation of the D’amico risk group classification for predicting survival following radical prostatectomy. J Urol. 2008;179(4):1354–60. discussion 60 – 1. 18289596 10.1016/j.juro.2007.11.061

[6] Ploussard G, Manceau C, Beauval JB, Lesourd M, Almeras C, Gautier JR, et al. Decreased accuracy of the prostate cancer EAU risk group classification in the era of imaging-guided diagnostic pathway: proposal for a new classification based on MRI-targeted biopsies and early oncologic outcomes after surgery. World J Urol. 2020;38(10):2493–500. 31838560 10.1007/s00345-019-03053-6

[7] EAU Guidelines. Edn. presented at the EAU Annual Congress Milan 2023. Treatment of high-risk localised disease EAU Guidelines Office, Arnhem, The Netherlands.

[8] ProZIB. ProZIB: evaluatie van de kwaliteit van prostaatkankerzorg: Netherlands comprehensive cancer organisation. 2019 [Available from: https://iknl.nl/projecten/prozib].

[9] Heesterman BL, Aben KKH, de Jong IJ, Pos FJ, van der Hel OL. Radical prostatectomy versus external beam radiotherapy with androgen deprivation therapy for high-risk prostate cancer: a systematic review. BMC Cancer. 2023;23(1):398. 37142955 10.1186/s12885-023-10842-1 PMC10157926

[10] Andic F, Izol V, Gokcay S, Arslantas HS, Bayazit Y, Coskun H, et al. Definitive external-beam radiotherapy versus radical prostatectomy in clinically localized high-risk prostate cancer: a retrospective study. BMC Urol. 2019;19(1):3. 30611260 10.1186/s12894-018-0432-6 PMC6321694

[11] Gunnarsson O, Schelin S, Brudin L, Carlsson S, Damber JE. Triple treatment of high-risk prostate cancer. A matched cohort study with up to 19 years follow-up comparing survival outcomes after triple treatment and treatment with hormones and radiotherapy. Scand J Urol. 2019;53(2–3):102–8. 30990112 10.1080/21681805.2019.1600580

[12] Hoffman KE, Penson DF, Zhao Z, Huang LC, Conwill R, Laviana AA, et al. Patient-reported outcomes through 5 years for active surveillance, surgery, Brachytherapy, or External Beam Radiation with or without androgen deprivation therapy for localized prostate Cancer. JAMA. 2020;323(2):149–63. 31935027 10.1001/jama.2019.20675 PMC6990712

[13] Koo KC, Cho JS, Bang WJ, Lee SH, Cho SY, Kim SI, et al. Cancer-Specific Mortality among Korean men with localized or locally advanced prostate Cancer treated with radical Prostatectomy Versus Radiotherapy: a Multi-center Study using Propensity Scoring and competing risk regression analyses. Cancer Res Treat. 2018;50(1):129–37. 28279064 10.4143/crt.2017.004 PMC5784628

[14] Reichard CA, Hoffman KE, Tang C, Williams SB, Allen PK, Achim MF, et al. Radical prostatectomy or radiotherapy for high- and very high-risk prostate cancer: a multidisciplinary prostate cancer clinic experience of patients eligible for either treatment. BJU Int. 2019;124(5):811–9. 31009137 10.1111/bju.14780

[15] Tward JD, O’Neil B, Boucher K, Kokeny K, Lowrance WT, Lloyd S, et al. Metastasis, mortality, and Quality of Life for Men with NCCN High and very high risk localized prostate Cancer after Surgical and/or combined modality Radiotherapy. Clin Genitourin Cancer. 2020;18(4):274–e835. 32335059 10.1016/j.clgc.2019.11.023

[16] Ciezki JP, Weller M, Reddy CA, Kittel J, Singh H, Tendulkar R, et al. A comparison between low-dose-rate Brachytherapy with or without Androgen Deprivation, External Beam Radiation Therapy with or without Androgen Deprivation, and Radical Prostatectomy with or without adjuvant or salvage Radiation Therapy for high-risk prostate Cancer. Int J Radiat Oncol Biol Phys. 2017;97(5):962–75. 28333019 10.1016/j.ijrobp.2016.12.014

[17] ClinicalTrials.gov. High-Risk prostatE cancer radiatiOn versus surgERy (Recover) [Available from: https://clinicaltrials.gov/study/NCT05931419].

[18] Dutch Association for Urology (Nederlandse Vereniging voor Urologie: NVU). Kwaliteitsnormen prostaatcarcinoom versie 4. 2018.

[19] Paner GP, Stadler WM, Hansel DE, Montironi R, Lin DW, Amin MB. Updates in the Eighth Edition of the Tumor-Node-Metastasis staging classification for urologic cancers. Eur Urol. 2018;73(4):560–9. 29325693 10.1016/j.eururo.2017.12.018

[20] van der Poel H, van der Kwast T, Aben K, Mottet N, Mason M. Imaging and T category for prostate Cancer in the 8th Edition of the Union for International Cancer Control TNM classification. Eur Urol Oncol. 2020;3(5):563–4. 31307959 10.1016/j.euo.2019.06.001

[21] Wei JT, Dunn RL, Litwin MS, Sandler HM, Sanda MG. Development and validation of the expanded prostate cancer index composite (EPIC) for comprehensive assessment of health-related quality of life in men with prostate cancer. Urology. 2000;56(6):899–905. 11113727 10.1016/s0090-4295(00)00858-x

[22] Aaronson NK, Ahmedzai S, Bergman B, Bullinger M, Cull A, Duez NJ, et al. The European Organization for Research and Treatment of Cancer QLQ-C30: a quality-of-life instrument for use in international clinical trials in oncology. JNCI: J Natl Cancer Inst. 1993;85(5):365–76. 8433390 10.1093/jnci/85.5.365

[23] Schmidt S, Garin O, Pardo Y, Valderas JM, Alonso J, Rebollo P, et al. Assessing quality of life in patients with prostate cancer: a systematic and standardized comparison of available instruments. Qual Life Res. 2014;23(8):2169–81. 24748557 10.1007/s11136-014-0678-8 PMC4155169

[24] Skolarus TA, Dunn RL, Sanda MG, Chang P, Greenfield TK, Litwin MS, et al. Minimally important difference for the expanded prostate Cancer Index Composite Short Form. Urology. 2015;85(1):101–5. 25530370 10.1016/j.urology.2014.08.044 PMC4274392

[25] Musoro JZ, Coens C, Sprangers MAG, Brandberg Y, Groenvold M, Flechtner HH, et al. Minimally important differences for interpreting EORTC QLQ-C30 change scores over time: a synthesis across 21 clinical trials involving nine different cancer types. Eur J Cancer. 2023;188:171–82. 37257278 10.1016/j.ejca.2023.04.027

[26] Sangha O, Stucki G, Liang MH, Fossel AH, Katz JN. The self-administered Comorbidity Questionnaire: a new method to assess comorbidity for clinical and health services research. Arthritis Rheum. 2003;49(2):156–63. 12687505 10.1002/art.10993

[27] Bouwmans C, Krol M, Severens H, Koopmanschap M, Brouwer W, Hakkaart-van Roijen L. The iMTA Productivity cost questionnaire: a standardized instrument for Measuring and Valuing Health-related Productivity losses. Value Health. 2015;18(6):753–8. 26409601 10.1016/j.jval.2015.05.009

[28] Profiel:. Wat kanker met u doet [Available from: https://www.profielstudie.nl/wat-doen-we/].

[29] van de Poll-Franse LV, Horevoorts N, van Eenbergen M, Denollet J, Roukema JA, Aaronson NK, et al. The patient reported outcomes following initial treatment and long term evaluation of Survivorship registry: scope, rationale and design of an infrastructure for the study of physical and psychosocial outcomes in cancer survivorship cohorts. Eur J Cancer. 2011;47(14):2188–94. 21621408 10.1016/j.ejca.2011.04.034

[30] Austin PC. An introduction to Propensity score methods for reducing the effects of confounding in Observational studies. Multivar Behav Res. 2011;46(3):399–424. 10.1080/00273171.2011.568786 PMC3144483 21818162

[31] Rosenbaum PR, Rubin DB. The central role of the propensity score in observational studies for causal effects. Biometrika. 1983;70(1):41–55.

[32] Robins J. A new approach to causal inference in mortality studies with a sustained exposure period—application to control of the healthy worker survivor effect. Math Modelling. 1986;7(9):1393–512.

[33] Snowden JM, Rose S, Mortimer KM. Implementation of G-computation on a simulated data set: demonstration of a causal inference technique. Am J Epidemiol. 2011;173(7):731–8. 21415029 10.1093/aje/kwq472 PMC3105284

[34] Jakobsen JC, Gluud C, Wetterslev J, Winkel P. When and how should multiple imputation be used for handling missing data in randomised clinical trials - a practical guide with flowcharts. BMC Med Res Methodol. 2017;17(1):162. 29207961 10.1186/s12874-017-0442-1 PMC5717805

[35] Sterne JA, White IR, Carlin JB, Spratt M, Royston P, Kenward MG, et al. Multiple imputation for missing data in epidemiological and clinical research: potential and pitfalls. BMJ. 2009;338:b2393. 19564179 10.1136/bmj.b2393 PMC2714692

[36] Penson DF. An update on randomized clinical trials in localized and locoregional prostate cancer. Urol Oncol. 2005;23(4):280–8. 16018945 10.1016/j.urolonc.2005.05.006

[37] Collaborators P. Early closure of a randomized controlled trial of three treatment approaches to early localised prostate cancer: the MRC PR06 trial. BJU Int. 2004;94(9):1400–1. 15610132 10.1111/j.1464-410X.2004.05224_3.x

[38] Wallace K, Fleshner N, Jewett M, Basiuk J, Crook J. Impact of a multi-disciplinary patient education session on accrual to a difficult clinical trial: the Toronto experience with the surgical prostatectomy versus interstitial radiation intervention trial. J Clin Oncol. 2006;24(25):4158–62. 16943531 10.1200/JCO.2006.06.3875

[39] Ritchie A, et al. Early closure of a randomized controlled trial of three treatment approaches to early localised prostate cancer: the MRC PR06 trial. BJU Int. 2004;94(9):1400–1. 15610132 10.1111/j.1464-410X.2004.05224_3.x

[40] Concato J, Lawler EV, Lew RA, Gaziano JM, Aslan M, Huang GD. Observational methods in comparative effectiveness research. Am J Med. 2010;123(12 Suppl 1):e16–23. 21184862 10.1016/j.amjmed.2010.10.004

[41] Yang W, Zilov A, Soewondo P, Bech OM, Sekkal F, Home PD. Observational studies: going beyond the boundaries of randomized controlled trials. Diabetes Res Clin Pract. 2010;88(Suppl 1):S3–9. 20466165 10.1016/S0168-8227(10)70002-4

[42] Lobo FS, Wagner S, Gross CR, Schommer JC. Addressing the issue of channeling bias in observational studies with propensity scores analysis. Res Social Adm Pharm. 2006;2(1):143–51. 17138506 10.1016/j.sapharm.2005.12.001

[43] Barocas DA, Chen V, Cooperberg M, Goodman M, Graff JJ, Greenfield S, et al. Using a population-based observational cohort study to address difficult comparative effectiveness research questions: the CEASAR study. J Comp Eff Res. 2013;2(4):445–60. 24236685 10.2217/cer.13.34 PMC4920086

[44] Kang R, Goodney PP, Wong SL. Importance of cost-effectiveness and value in cancer care and healthcare policy. J Surg Oncol. 2016;114(3):275–80. 27334052 10.1002/jso.24331 PMC5048466

